# A restrospective review of fixed drug eruptions presenting at a tertiary dermatology centre in Singapore between 2008 and 2012

**DOI:** 10.1186/2045-7022-4-S3-P77

**Published:** 2014-07-18

**Authors:** Yee kiat heng, Yik Weng Yew, Derek Lim, Yen Loo Lim

**Affiliations:** 1 National Skin Centre, Singapore; 2 National Skin Centre, Dermatology, Singapore; 3 National University of Singapore, Yong Loo Lin School of Medicine, Singapore; 4 National Skin Centre, Dermatology, Singapore

## Introduction

Fixed drug eruption (FDE) is a well-recognised localized cutaneous adverse drug reaction, characterized by its recurrence in the same sites upon re-exposure to the causative drug. We aimed to better understand the epidemiology, clinical features as well as the causative drugs of fixed drug eruptions in Singapore.

## Methods

A retrospective chart review of all cases with suspected FDE seen in National Skin Centre, Singapore between 2008 and 2012 was performed. All cases were classified into whether the diagnosis of FDE was definite, probable, possible or unlikely based on WHO-UMC Causality Assessment criteria. Patients were classified as probable FDE if there was typical cutaneous eruption which was temporally related to exposure to a clearly-identified drug and not attributable to other causes. Definite cases had confirmation with positive drug patch test or provocation test.

## Results

There were 126 patients who were seen for suspected FDE. Patients were evaluated with history-taking and physical examination. Skin biopsy or drug patch or provocation tests were performed when indicated in consenting patients. Almost half of the cases (46.0%) had definite or probable diagnosis of FDE with identification of the causative drug. Seven percent of patients were considered unlikely to have FDE, while 46.8% of the patients had possible diagnosis of FDE for which the causative drug could not be determined or the patient declined further evaluation. Of the 58 patients with definite or probable FDE, 70.7% presented with more than 1 site of involvement and 20.7% had genital lesions. Two patients had positive drug patch tests while 7 patients had positive drug provocation tests. Four patients had drug provocation tests to exclude other possible causative drugs. The most common causative drugs were non-steroidal anti-inflammatory drugs (NSAIDs), paracetamol and doxycycline. FDE was caused by anti-histamines in 4 cases, with 1 case having positive patch test to both hydroxyzine and cetirizine.

## Conclusion

It is a well-recognised fact that FDE may be caused by NSAIDs, paracetamol and doxycycline. Clinicians should also be aware that other commonly used drugs such as antihistamines, which are often thought to be safe, may occasionally cause FDE. Cross-reactivity between various antihistamines in causing FDE may also seen. Drug patch or provocation tests should be considered for complete evaluation.

